# Using Mind-Body Medicine to Reduce the Long-Term Health Impacts of COVID-Specific Chronic Stress

**DOI:** 10.3389/fpsyt.2021.585952

**Published:** 2021-02-22

**Authors:** Vojislav Maric, Jyoti Mishra, Dhakshin S. Ramanathan

**Affiliations:** ^1^Neural Engineering and Translation Labs, Department of Psychiatry, University of California, San Diego, La Jolla, CA, United States; ^2^Department of Mental Health, Veteran's Affairs San Diego Medical Center, San Diego, CA, United States

**Keywords:** chronic stress, mindfulness, exercise, relaxation response, COVID-19

## Introduction

Mental health continues to be a rising concern for the global population during the COVID-19 pandemic (1, 2). Addressing these issues requires us to consider the COVID-19 pandemic as a major, global and chronic life-stressor. Stresses related to the pandemic include the risk/fear of getting infected; social isolation; lack of schooling for children; potential for increased interpersonal conflict (domestic violence/trauma), and job/income loss (1, 2). While exact numbers are difficult to come by, early evidence suggests that the global COVID-19 pandemic has led to an increase in acute stress, along with a corollary increase in anxiety and depression across multiple countries and populations studied (1, 3–5). However, these stressors have already lasted for almost a year and may continue as lockdowns continue to be enforced worldwide, thus making them chronic rather than acute in nature. Moreover, despite the advent of vaccines, public health experts, such as Dr. Fauci (head of the US National Institute of Allergy and Infection Disease) suggested a return to normality might only occur at the end of 2021 in the US. Viewing the effects of COVID-19 pandemic as a chronic stressor is important for two reasons: (1) it suggests that the long-term effect of pandemic-related stress may be a worsening of both chronic physical and mental health, both of which are well-documented related to the impacts of chronic stress (6–8). (2) It suggests that specific, practical, affordable, and globally implementable strategies shown to help with chronic stress can be utilized.

## Physiologic Effects of Chronic Stress

Stress can be considered both acute and chronic (7, 8). Acute stress (as occurs when facing a short-lived life-threatening situation) triggers a stress response that includes an increase in catecholamine and glucocorticoid “stress” hormones from the adrenal gland along with an increase in activity of the sympathetic nervous system (7). Chronic, repeated stress, as is currently occurring due to the COVID-19 pandemic, leads to a sustained elevation of this stress response. A chronic increase in catecholamines results in a long-term increase in heart rate and blood pressure along with disruptions of the circadian system regulating sleep (6). Dysregulation of stress hormones driven by chronic stress can lead to both over and under-active glucocorticoid systems, further impacting a number of biological systems. These disruptions can lead to a number of physiological effects, such as an increased desire for high fat/salt foods (6, 9), worsening cardiovascular risk factors (hyperlipidemia, pro-inflammatory state) and impaired brain function, including dysfunction of the hippocampus and prefrontal cortex and increased growth of parts of the amygdala directly linked with anxiety (8). As a consequence of this litany of biological effects, chronic stress has been linked with worse mental health (depression, anxiety disorders, PTSD, and substance abuse) and physical health problems (insulin resistance, diabetes, and cardiovascular disease outcomes most prominently) (6, 9). Given all of this, it is important to recognize that the increase in stress experienced globally during this pandemic may lead to a long-term increase in both physical and mental health sequelae including worsening cardiovascular risk factors, anxiety, and depression (6, 9).

## Mind-Body Medicine as a Key Tool to Mitigate Chronic Stress

The Centers for Disease Control and Prevention (CDC), one of the major public health governing bodies in America, and others, have recommended common-sense strategies focused on mental health well-being during the COVID-19 pandemic (https://www.cdc.gov/coronavirus/2019-ncov/daily-life-coping/managing-stress-anxiety.html). Many of these mind-body medicine approaches have shown clear efficacy in improving the psychological and physiological manifestations of chronic stress. From this perspective, such approaches should be considered an essential first-line response to dealing with pandemic-related chronic stress for all. While mind-body medicine can cover a range of practices, most of them can be grouped into a category of practices that trigger the “relaxation response,” a physiological state of lower sympathetic tone, increased parasympathetic tone, and lower resting heart rate, respiratory rate and blood pressure (10), essentially the opposite of the “flight-fight” response. Three practices also fit important criteria for implementation (low-cost, scalable, globally implementable, and evidence-based): physical practices (e.g., exercise), mindfulness-based practices (meditation, respiratory control), and practices focused on increasing social engagement. Wide-spread adoption of these practices would be safe, cost-effective, evidence-based methods of promoting resilience to chronic stress during the COVID-19 pandemic and to potentially prevent the development of long-term mental/physical ill-health symptoms.

### Exercise

Exercise counteracts many of the physical manifestations of stress associated with increased cardiovascular risk (blood pressure, insulin resistance, lipids) (9, 11). In addition, exercise has been shown to diminish the psychological impacts of stress: it improves mood and affect, diminishes anxiety and diminishes reports of perceived stress (11). Systematic meta-analyses (9, 10), have shown that exercise has at least a moderate effect size in treating anxiety, depression and other stress-related mental health disorders, with effect sizes similar to that observed with pharmacotherapy and psychotherapy. Thus, exercise should be recommended as an effective stress-reduction tool to help individuals cope with mild to moderate psychiatric anxiety and mood symptoms emerging as a consequence of the COVID-19 pandemic.

### Mindfulness

Despite the many observed physical manifestations of chronic stress, it is probably most clearly felt as a psychological state of perturbation, anxiety or distress. For this reason, mindfulness approaches have long been seen as a specific antidote to stress (12). Like exercise, mindfulness practices seem to improve both the physical and psychological impacts of chronic stress, including blood pressure, heart rate, cortisol, CRP (a marker of inflammation) and lipids (13). These effects may be due to the “relaxation response” described above. Mindfulness-based approaches seem to be about as effective as other evidence-based treatments for many psychiatric conditions linked with chronic stress, including depression, pain, smoking and addictive disorders (14). Interestingly, part of the effects of meditation may be linked with slowing of the breath. Simple nasal diaphragmatic breathing with awareness is an effective mechanism for leading to the relaxation response (15, 16) with powerful effects on core brain circuits (parasympathetic nervous system, prefrontal cortex) involved in mood and stress regulation (17). These effects have been observed for a long time: the *Hatha Yoga Pradipika* text from the fifteenth century states: “When the breath is irregular, the mind wavers; when the breath is steady, so is the mind. To attain steadiness, the yogi should restrain (i.e., slow) his breath” (18).

### Social Engagement

Social isolation is known to be a unique but important stressor (8). It has been demonstrated that social isolation is a known stressor that is associated with negative health outcomes of all ages (19). While the above methods (exercise and mindfulness practices) may help with this stressor, maintaining social ties (both “strong” and “weak”), at least in certain populations, can also be an important aspect of dealing with chronic stress and maintaining resilience (20). Recent social media research shows that strong ties can be maintained globally across continents on online platforms (21). Thus, even with health recommendations for physical distancing, social engagement is possible with current online technologies, though excessive use can also cause harm, especially in adolescents (22, 23).

## Practical Recommendations and Discussion

With all of the practices above (physical exercise, meditation/respiratory control, social engagement), we believe specific recommendations can be made, based on evidence, to promote stress resilience. For exercise: at least 10–15 min/day of moderate intensity exercise can promote stress resilience. It is less clear if the type of exercise matters, and neither extended (i.e. longer than 30 minutes) or high intensity exercise seems necessary for stress mitigation (24, 25), For mindfulness: it is unclear if the type of meditation matters. The simplest form of meditation is breath awareness, and this, coupled with intranasal slow respirations to trigger the relaxation response, will likely be as effective as any more complicated meditation style. Moreover, meditation ~5 × /week, for as short as 15 min/day, may be sufficient to deliver effects (26). In Pranayama, one of most commonly practiced breath control techniques, slowing the breath arises by maintaining standardized pauses during and between inspiration and expiration cycles and using nostril breathing. Slowing the breath, using a beginner” Pranayama respiration exercise uses a 1:1:1 cycle of inspiration, starting with 4 s inhale, 4 s pause, and a 4 s exhale, can further leverage direct respiratory coupling to mood regulation circuits. Even during COVID-19-pandemic related physical distancing recommendations, digital apps may be used to further support mental health and even to enhance adherence to practice (27, 28). Social support is known to enhance intervention adherence (29, 30), hence, it could be ideal to leverage online social group support simultaneous with physical exercise and mindfulness activities.

## Author Contributions

JM, VM, and DR jointly conceptualized and wrote the manuscript. All authors contributed to the article and approved the submitted version.

## Conflict of Interest

The authors declare that the research was conducted in the absence of any commercial or financial relationships that could be construed as a potential conflict of interest.
